# Neonatal exposure to hypoxia induces early arterial stiffening via activation of lysyl oxidases

**DOI:** 10.14814/phy2.15656

**Published:** 2023-04-11

**Authors:** Jochen Steppan, Kavitha Nandakumar, Huilei Wang, Rosie Jang, Logan Smith, Sara Kang, William Savage, Maria Bauer, Rira Choi, Travis Brady, Bulouere Princess Wodu, Susanna Scafidi, Joseph Scafidi, Lakshmi Santhanam

**Affiliations:** ^1^ Department of Anesthesiology and Critical Care Medicine Johns Hopkins University School of Medicine, Kennedy Krieger Institute Baltimore Maryland USA; ^2^ Department of Biomedical Engineering Johns Hopkins University School of Medicine, Kennedy Krieger Institute Baltimore Maryland USA; ^3^ Department of Chemical and Biomolecular Engineering Johns Hopkins University School of Medicine, Kennedy Krieger Institute Baltimore Maryland USA; ^4^ Department of Neurology Johns Hopkins University School of Medicine, Kennedy Krieger Institute Baltimore Maryland USA; ^5^ Department of Pediatrics Johns Hopkins University School of Medicine, Kennedy Krieger Institute Baltimore Maryland USA; ^6^ Michael V. Johnston Center for Developmental Neuroscience Kennedy Krieger Institute Baltimore Maryland USA

## Abstract

Hypoxia in the neonatal period is associated with early manifestations of adverse cardiovascular health in adulthood including higher risk of hypertension and atherosclerosis. We hypothesize that this occurs due to activation of lysyl oxidases (LOXs) and the remodeling of the large conduit vessels, leading to early arterial stiffening. Newborn C57Bl/6 mice were exposed to hypoxia (FiO_2_ = 11.5%) from postnatal day 1 (P1) to postnatal day 11 (P11), followed by resumption of normoxia. Controls were maintained in normoxia. Using in vivo (pulse wave velocity; PWV) and ex vivo (tensile testing) arterial stiffness indexes, we determined that mice exposed to neonatal hypoxia had significantly higher arterial stiffness compared with normoxia controls by young adulthood (P60), and it increased further by P120. Echocardiography performed at P60 showed that mice exposed to hypoxia displayed a compensated dilated cardiomyopathy. Western blotting revelated that neonatal hypoxia accelerated age‐related increase in LOXL2 protein expression in the aorta and elevated LOXL2 expression in the PA at P11 with a delayed decay toward normoxic controls. In the heart and lung, gene and protein expression of LOX/LOXL2 were upregulated at P11, with a delayed decay when compared to normoxic controls. Neonatal hypoxia results in a significant increase in arterial stiffness in early adulthood due to aberrant LOX/LOXL2 expression. This suggests an acceleration in the mechanical decline of the cardiovascular system, that contributes to increased risk of hypertension in young adults exposed to neonatal hypoxia that may increase susceptibility to further insults.

## Graphical abstract

Neonatal hypoxia accelerates arterial aging through lysyl oxidase‐like 2 activation in both systemic and pulmonary circulation. Created with BioRender.com.

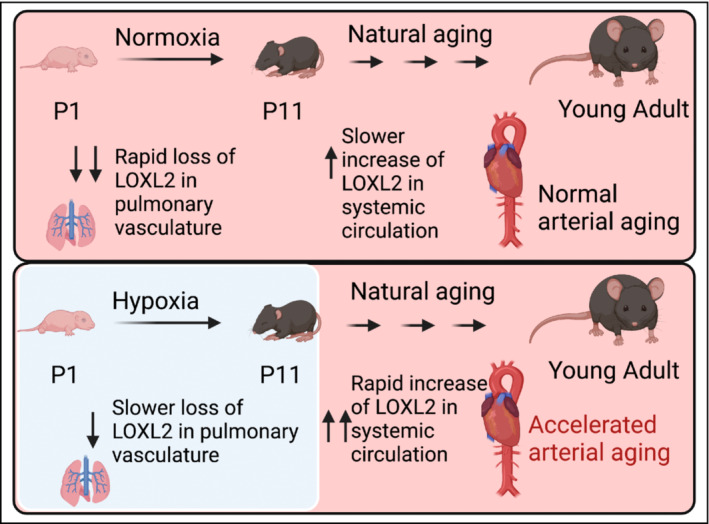

## 1 INTRODUCTION

Neonatal hypoxia adversely impacts cardiovascular development, resulting in growth retardation, atherosclerosis, the development of hypertension, and susceptibility to myocardial ischemia in adulthood (Bates et al., 2020; Lewandowski et al., 2020; Netuka et al., 2006). Chronic hypoxia in early life can occur due to several reasons. For example, neonates born extremely preterm are highly susceptible to chronic hypoxemia during the neonatal period because of preterm lung disease (bronchoplumonary dysplasia). Chronic hypoxemia during the neonatal period is also evident in neonates with unrepaired cyanotic congenital heart disease and other complex congenital heart diseases. Despite these early life challenges, many of these neonates survive into adulthood due to advances in neonatal care. A significant amount of research has focused on the effects of neonatal hypoxia on neurodevelopmental and cognitive outcomes over time, and mechanisms contributing to these morbidities. However, there is limited mechanistic understanding regarding the effect of neonatal hypoxemia on vascular development and long‐term outcome. Recent large epidemiological studies demonstrated a higher incidence of adverse cardiovascular health in adulthood including a higher risk of hypertension, ischemic heart disease, atherosclerosis, and stroke in these children, even though the hypoxia exposure resolves in childhood (Crump et al., 2011; de Jong et al., 2012; Johansson et al., 2005; Raju et al., 2017; Saigal & Doyle, 2008; Skudder‐Hill et al., 2019). The emergence of cardiovascular disease in adulthood is likely connected to “developmental programming.” (Ostadal et al., 2014; Soukhova‐O'Hare et al., 2008) However, to date we have a dearth of understanding regarding the changes during early life that result in this pathology and phenotype.

A potential culprit in this development is vascular stiffening, which has been shown to underlie the development of aging‐associated systolic hypertension and atherosclerosis (Jung et al., 2013; Oh et al., 2017; Steppan et al., 2011, 2012, 2014, 2016, 2017, 2019, 2020). The remodeling of the vascular matrix is a significant component of vascular stiffening in aging. While hypoxia is known to stimulate remodeling of the microvasculature, for example, tumor angiogenesis (Chen et al., 2009; Muz et al., 2015), pulmonary vasculature in pulmonary hypertension (Ball et al., 2014; Pak et al., 2007), and formation of collateral vessels in ischemia‐associated hypoxia (Aghajanian et al., 2021; Zhang et al., 2020), little is known about the response of large compliance vessels, particularly in neonates. In the context of vascular matrix remodeling, lysyl oxidases (LOXs) emerge as an important class of enzymes. LOXs are amine oxidases that establish and remodel extracellular matrices by catalyzing crosslinking of collagen fibers. Prior studies have clearly demonstrated hypoxia‐induced expression of LOX and LOXL2, mediated at least in part by hypoxia‐induced factor 1‐alpha (HIF1ɑ). (Besiktepe et al., 2017; Chen et al., 2020; Gundemir et al., 1833; Kumar & Mehta, 2012; Wang et al., 2017) We have previously shown that LOXL2 activation promotes vascular stiffening associated with aging. (Steppan et al., 2019; Wang, Poe, et al., 2021) In addition, studies have established LOXL2 as a key factor in many fibrotic diseases involving an element of a hypoxic microenvironment including interstitial cardiac fibrosis (Yang et al., 2016), pulmonary fibrosis (Chien et al., 2014), and pulmonary hypertension. (Nave et al., 2014) Importantly, prototypical LOX and lysyl oxidase‐like 2 (LOXL2) are shown to be essential for vasculogenesis and development of the cardiovascular system. (Bignon et al., 2011; Chen et al., 2020; Peng et al., 2019; Steppan et al., 2019; Wang et al., 2017; Zaffryar‐Eilot et al., 2013) Prior studies have established a rapid loss of LOX/LOXL2 expression in the vasculature in the postnatal period.

In this study, we tested the hypothesis that neonatal exposure to hypoxia is associated with accelerated aortic stiffening in early adulthood and examined LOX/LOXL2 activation as a putative underlying mechanism. We further postulated that vascular stiffening is widespread, occurring not just in the aorta, but also in the pulmonary vessels, thus promoting accelerated aging‐associated arterial stiffening and increasing the risk for cardiopulmonary disease.

## 2 METHODS

### 2.1 Animals

Male and female C57BL/6J mice were used in this study. Mice were bred and housed at the Johns Hopkins University School of Medicine animal care facility. All protocols were approved by the Institutional Animal Care and Use Committee. The animals were fed and watered ad libitum and maintained on a 12‐h light/dark cycle.

### 2.2 Neonatal hypoxia model

**FIGURE 1:**
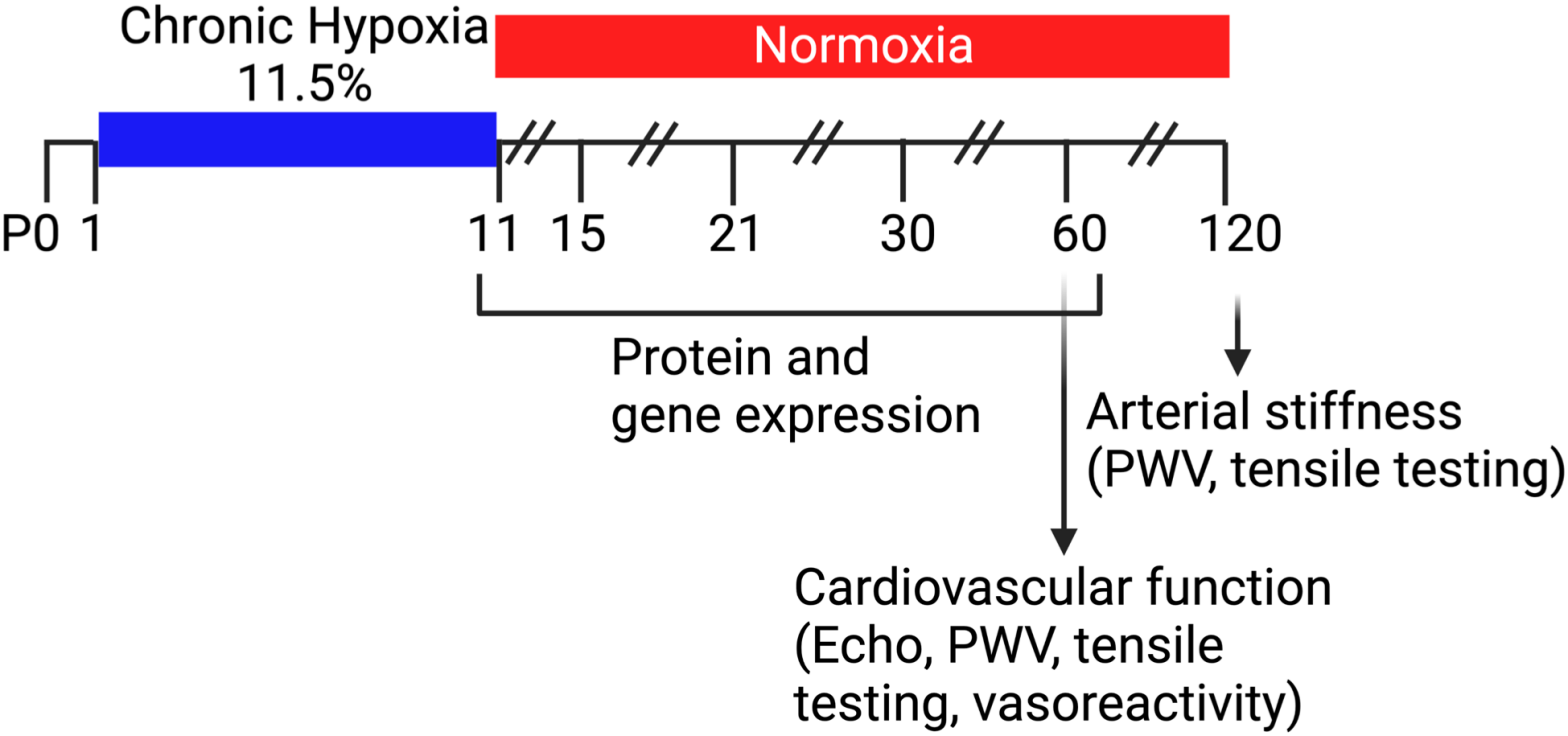
Schematic representation of the experimental timeline. Created with BioRender.com.

Mice were randomly assigned to either undergo hypoxic rearing or serve as normoxic controls. Litters were culled to no more than six pups per dam (Figure 1). Mice pups were kept with their mothers and with a foster mother. Mice pups assigned to undergo hypoxia were placed in a sealed chamber within 12 h of birth where they remained with their mothers and foster mothers from postnatal day 1 (P1) until postnatal day 11 (P11). This age was chosen because in the mouse, brain, and cardiovascular development mimics the third trimester of gestation for human development. The oxygen levels in a sealed hypoxia chamber were maintained by computer controller at a FiO_2_ of 11.5% (Biospherix), which results in oxygen saturations (SaO_2_) of approximately 10% less than normoxia controls and PaO2s between 60 and 70s (Kline et al., 1998; Lee et al., 2009). The oxygen content was maintained at 11.5% by displacement with nitrogen gas as previously described. (Scafidi et al., 2014) At P11, mice were removed from the chamber and kept in normoxic conditions (FiO_2_ = 21%). Mice were sacrificed at postnatal days 11, 15 (P15; “young”/infancy), 21 (P21; weaning from lactating dam with more complex activities requiring oxygenation and perfusion), 30 (P30; adolescence with a large growth spurt leading to sexual maturity), 60 (P60; young, sexually mature adult), and 120 (P120; adulthood wherein early arterial stiffening would be evident). Heart, aorta, lung, and main pulmonary artery (PA) were dissected out and used for experiments described below. At P60, pulse wave velocity (PWV), BP, and echo analyses were performed prior to sacrifice and extraction of the aorta for further analysis. At P120, PWV and BP were measured, and then, the aorta was extracted for ex vivo analysis. A total of 22 dams (normoxia) and 24 dams (hypoxia) were used for this study.

### 2.3 *Lox* and *Loxl2* gene expression analysis

RNA was extracted from lung and heart using Aurum™ Total RNA Mini Kit (Bio‐Rad) and reverse transcribed using iScript™ cDNA Synthesis Kit (Bio‐Rad). The expression of *Lox* and *Loxl2* genes was analyzed by real‐time PCR. 18S was used as a control. Primers used were as follows: *Lox* forward 5′‐atgggtaccacaggcgcttt‐3′ and reverse 5′‐ gtctgccgcataggtgtcat −3′; *Loxl2* forward 5′‐attaaccccaactatgaagtgcc‐3′ and reverse 5′‐ ctgtctcctcactgaaggctc‐3′; 18S forward 5′‐gcaattattccccatgaacg‐3′ and reverse 5′‐ ggcctcactaaaccatccaa‐3′. The relative expression of *Lox* and *Loxl2* genes was calculated using the ΔΔCt method, with P11 normoxia as the control.

### 2.4 Western blotting

LOX and LOXL2 protein abundance in heart, lung, aorta, and main PA was determined by Western blotting. For sample preparation, heart and lung were crushed in liquid nitrogen and washed in PBS buffer to remove blood and then homogenized and sonicated in mammalian protein extraction reagent (M‐PER, Thermo Fisher). Aorta was crushed and directly lysed in M‐PER containing protease inhibitor cocktail (Roche). Tissue samples were then centrifuged to recover the soluble cytosol fraction and the insoluble matrix fraction. Protein concentration in the soluble cytosol fraction was determined by the Bradford assay (Bio‐Rad). The insoluble matrix fraction was directly solubilized into 1.5× Laemmli buffer with beta‐mercaptoethanol (BME) in a volume calculated based on cytosolic protein concentration (100 μL for a 2 mg/mL cytosol protein concentration). Owing to the small sample size, PA specimens were directly homogenized in 50 μL of 1.5× Laemmli buffer with BME; 25 μL were used for Western blotting. Samples were separated by SDS‐PAGE and electrotransferred onto nitrocellulose membrane. Membranes were blocked in 3% nonfat milk in TBST (Tris‐buffered saline, 0.1% Tween 20) and then incubated with primary antibody (1:1000, 2 h) and secondary antibody (1:10,000, 2 h). Membranes were washed in TBST and developed with the Clarity Western ECL system (Bio‐Rad). Antibodies used were as follows: LOX rabbit polyclonal (Thermo Fisher PA1‐16953), LOXL2 rabbit monoclonal (Abcam ab179810), HSC70 rabbit polyclonal antibody (Thermo Fisher PA5‐27337), goat anti‐mouse IgG (H + L)‐HRP conjugate (Bio‐Rad 1706516), and AffiniPure goat anti‐rabbit IgG (H + L)‐HRP conjugate (Jackson ImmunoResearch 111035144). Densitometry analyses were performed using ImageLab software (Bio‐Rad). Total protein determined by Ponceau S staining of the nitrocellulose membrane was used as loading control. Data were normalized to P11 normoxia LOX or LOXL2 protein abundance.

### 2.5 Blood pressure measurements

Blood pressures were measured using a tail‐cuff measurement system (Kent Scientific) (Steppan et al., 2014, 2020). Briefly, adult mice (P60) were acclimatized over 10 days prior to first measurement. Conscious animals were gently restrained in a translucent plastic cone with the tail exposed and a total of 10 measurements were obtained.

### 2.6 Pulse wave velocity (PWV)

PWV was measured noninvasively at P60 and P120 using a high‐frequency, high‐resolution Doppler spectrum analyzer, as in our previous studies (Steppan et al., 2019, 2020). Briefly, the mice were anesthetized with 1.5% isoflurane and placed supine on a heated electrode pad (37°C). We used a 10 MHz probe to record the aortic pulse wave at the thorax and abdomen separately at a distance of 2 cm. The electrocardiogram was recorded simultaneously and the time taken by the wave to travel between the thoracic and abdominal aorta was measured using the R wave of the electrocardiograph as a fixed point. PWV was calculated as the distance (in cm) divided by the pulse transit time (in s) between the two points.

### 2.7 Echocardiography

At P60, chest hair was removed using hair remover lotion. Animals were lightly anesthetized using isoflurane (1.5%) via nosecone and placed on a heating pad and core temperature was maintained at 37°C. A Vevo 2100 ultrasound machine (Visual Sonics) equipped with a 18–38 MHz mechanical transducer (MS400) was used to obtain the images needed to determine left ventricular volume, end‐diastolic volume, stroke volume, ejection fraction, cardiac output, left ventricular mass, global longitudinal strain, fractional shortening, and heart rate.

### 2.8 Tensile testing

The elastic properties of pulmonary artery rings were analyzed by tensile testing (Jung et al., 2013; Steppan et al., 2014, 2017). Briefly, at P60 and P120 the main pulmonary arteries and the aortas were harvested in a subset of animals and cut into 2‐mm rings. Three to four rings from each animal were tested. Transverse and longitudinal images of the sample were obtained to calculate vessel dimensions [lumen diameter (Di), wall thickness (t), and length (L)]. Samples were mounted on an electromechanical puller (DMT). After calibration, the pins were moved apart using an electromotor and displacement and force were recorded continuously. Engineering stress (S) was calculated by normalizing force (F) to the initial stress‐free area of the specimen (S = F/2 t × L; where t, thickness and L, length of the sample). Engineering strain (λ) was calculated as the ratio of displacement to the initial stress‐free diameter. Stress–strain relationships were represented by the equation S = α exp (βλ), where α and β are constants. α and β were determined by nonlinear regression for each sample and used to generate stress–strain curves by treating the x‐axis as a continuous variable.

### 2.9 Statistical analysis

Data are presented as mean ± standard error of the mean (SEM). Student's *t*‐test was used to compare two means. Sample size (*n*) is indicated for each reported value. For statistical evaluation, two means were compared by nonparametric Wilcoxon rank‐sum test and groups were compared with the Kruskal–Wallis nonparametric test with Dunn's comparison. For multiple comparisons, two‐way analysis of variance with Bonferroni post hoc analysis was used. Means were considered to be statistically different at *p* < 0.05.

## 3 RESULTS

### 3.1 Neonatal hypoxia exposure causes vascular stiffening and cardiomyopathy in early adulthood

**FIGURE 2:**
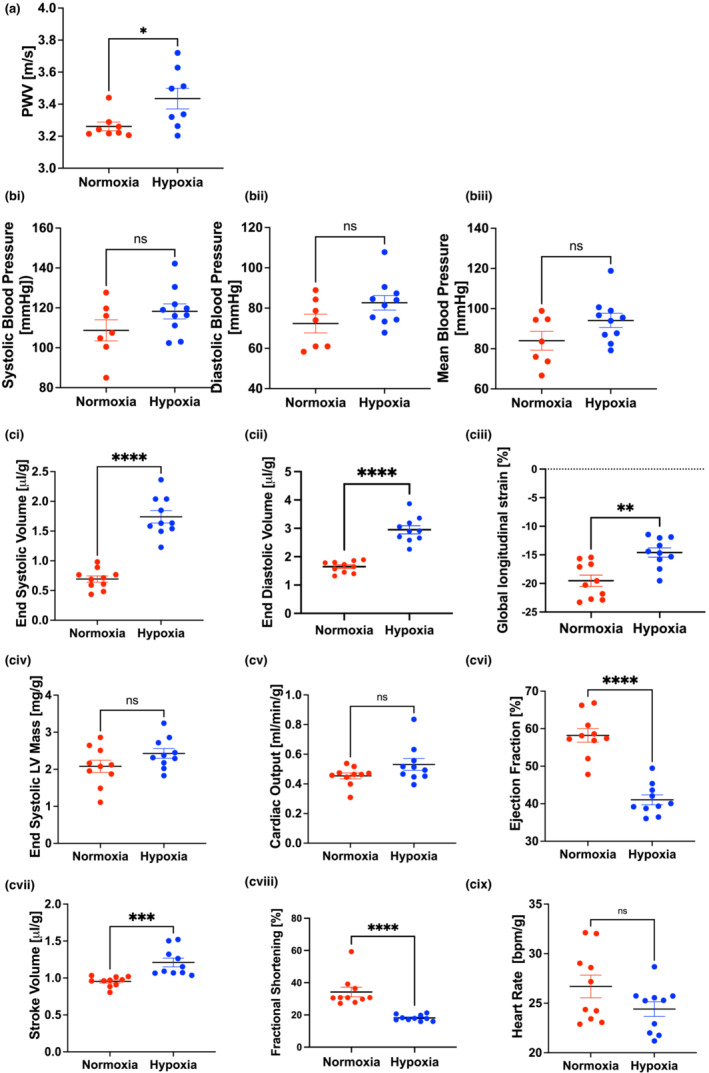
Neonatal hypoxia exposure results in increased aortic stiffness and a dilated cardiomyopathy in adult mice. (a) Aortic pulse wave velocity (PWV) in 60 day old mice that were exposed to hypoxia as a newborn (*n* = 8, *p* = 0.027; 4 males and 4 females). (b) systolic (bi), diastolic (bii), and mean blood pressure (biii) in P60 mice with and without neonatal hypoxia (systolic blood pressure: *p* = 0.15; diastolic blood pressure: *p* = 0.09; mean blood pressure: p, *p* = 0.10). (*n* = 7 for normoxia; 4 males and 3 females; *n* = 10 for hypoxia; 5 male and 4 female) (c) Echocardiography at P60 comparing mice exposed to neonatal hypoxia mice kept at room air (*n* = 10 per group; 5 male and 5 female): End‐systolic volume (ci) (*p* < 0.0001), end‐diastolic volume (cii) (*p* < 0.0001), and global longitudinal strain (ciii) (*p* < 0.01) are significantly higher in mice exposed to hypoxia during the newborn period with similar end systolic left ventricular (LV) mass (civ) (*p* = 0.058). There is no change in cardiac output at rest (cv) (*p* = 0.15), but ejection fraction (cvi) (*p* < 0.0001) is lower and stroke volume (cvii) (*p* = 0.005) is modestly elevated in mice exposed to hypoxia in the newborn period. Fractional shortening (cviii) (*p* < 0.001) is lower in mice exposed to hypoxia during the newborn period and heart rate (cix) is similar between the groups (*p* = 0.08). (*p < 0.05, **p < 0.01, ***p < 0.001, ****p < 0.0001, by unpaired *t*‐test).

Mice were reared in hypoxia (FiO2 11.5%) during the neonatal period (P1‐P11) and subsequently maintained in normoxia (FiO2 21%) until adulthood (Figure 1). We evaluated in vivo cardiovascular mechanics and function in early adulthood at P60. PWV, the gold‐standard index of in vivo vascular stiffness arising from both passive and active vascular properties, was significantly higher at P60 in mice that were exposed to hypoxia during the neonatal period when compared to normoxic controls (Figure 2a). Blood pressure was similar in the two groups (Figure 2bi–iii). Echocardiography demonstrated the presence of a compensated dilated left ventricular cardiomyopathy with an increased end‐systolic volume (Figure 2ci), increased end‐diastolic volume (Figure 2cii), elevated global longitudinal strain (Figure 2ciii), and a normal end systolic left ventricular mass (Figure 2civ) in the mice exposed to neonatal hypoxia when compared to normoxic controls. Cardiac output was preserved (Figure 2cv) despite a decreased ejection fraction (Figure 2cvi) and a modestly increased stroke volume (Figure 2cvii). Fractional shortening was lower in the hypoxia group (Figure 2vii) and heart rate was similar in the two groups (Figure 2cviii). Cardiac and vascular measures were independent of sex.

**FIGURE 3:**
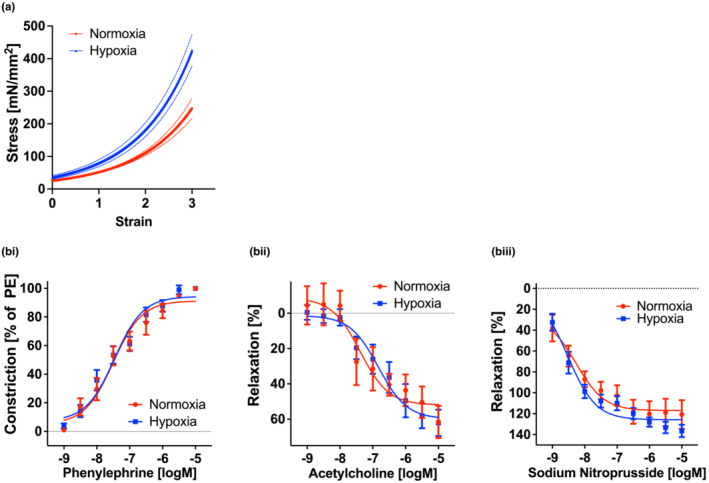
Neonatal hypoxia exposure leads to increased vascular stiffness and remodeling in adult mice without affecting vasoreactivity. (a) Tensile testing of P60 mouse aortas from normoxic and hypoxic neonates (*n* = 12; 6 males, 6 females; *p* < 0.0001). (b) Vascular reactivity (*n* = 6–10, *p* = 0.99; equal males and females) to phenylephrine (bi), the endothelial dependent vasodilatory response to acetylcholine (bii) (*p* = 0.98), and the nitric oxide independent response to sodium nitroprusside (biii) (*p* = 0.97). *p < 0.05, **p < 0.01, ***p < 0.001, ****p < 0.0001, by 2‐Way ANOVA with Bonferroni post hoc analysis.

Passive tensile testing of the aorta ex vivo revealed a marked increase in aortic stiffness in adult mice (P60) that were exposed to neonatal hypoxia (from P1‐P11) compared with control normoxia animals (Figure 3a). Active vascular properties, including the vasoconstrictive response to phenylephrine (Figure 3bi), endothelial‐dependent relaxation (Figure 3bii), and nitric oxide‐independent vasorelaxation (Figure 3biii), were similar in the two groups, indicating that vascular smooth muscle and endothelial function were unimpaired.

### 3.2 LOXL2 is the primary LOX that is upregulated in the aorta and heart of neonatal hypoxic exposure

**FIGURE 4:**
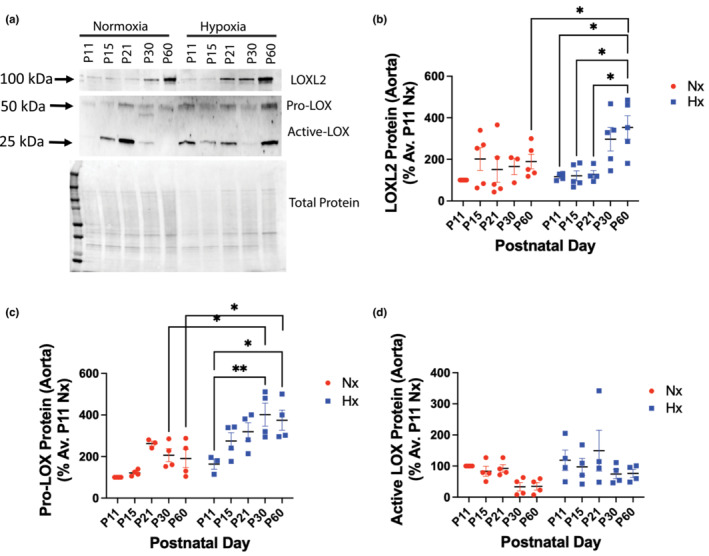
Aortic LOXL2 expression is elevated in adulthood after neonatal hypoxia exposure. (a) Representative Western blots for LOXL2, Pro‐LOX, and active‐LOX protein expression in the aortic ECM. Densitometry analysis of LOXL2 (b; 3 males, 2 females), Pro‐LOX (c; 2 males, 2 females), and active (processed; 2 males, 2 females) LOX (d). *p < 0.05, **p < 0.01, ***p < 0.001, ****p < 0.0001, by 2‐way ANOVA with Bonferroni post hoc analysis. Nx = normoxia, Hx = neonatal hypoxia from P1‐P11.

We next evaluated whether aortic stiffening and cardiac changes in the mice exposed to hypoxia in the neonatal period are due to the activation of LOXL2 and LOX, as both these enzymes are shown to be critical for vasculogenesis. (Bignon et al., 2011; Wei et al., 2020) Due to the small size of the aorta in juvenile mice, mRNA isolation and qPCR were not feasible. Thus, we performed Western blotting in the aorta to detect differences in LOX and LOXL2 protein expression. LOX is secreted as an inactive pro‐enzyme and is processed by BMPs to its active form. LOXL2, on the other hand, does not require processing and exhibits catalytic activity in the full‐length form. We noted a modest elevation in pro‐LOX and LOXL2 protein expression in the aorta in the hypoxic mice at P11, but this was not statistically significant (Figure 4a–c). Intriguingly, age‐associated increase in LOXL2 protein expression was accelerated in the neonatal hypoxia‐exposed group when compared to the normoxic controls. By P60, LOXL2 protein abundance in the aortic ECM was significantly higher in neonatal hypoxia‐exposed mice when compared to normoxic counterparts. Pro‐LOX also increased with age and by P30 was significantly higher in the neonatal hypoxia‐exposed group when compared to normoxic counterparts and was further increased by P60. Active LOX, however, was unchanged with age and hypoxia (Figure 4d).

**FIGURE 5:**
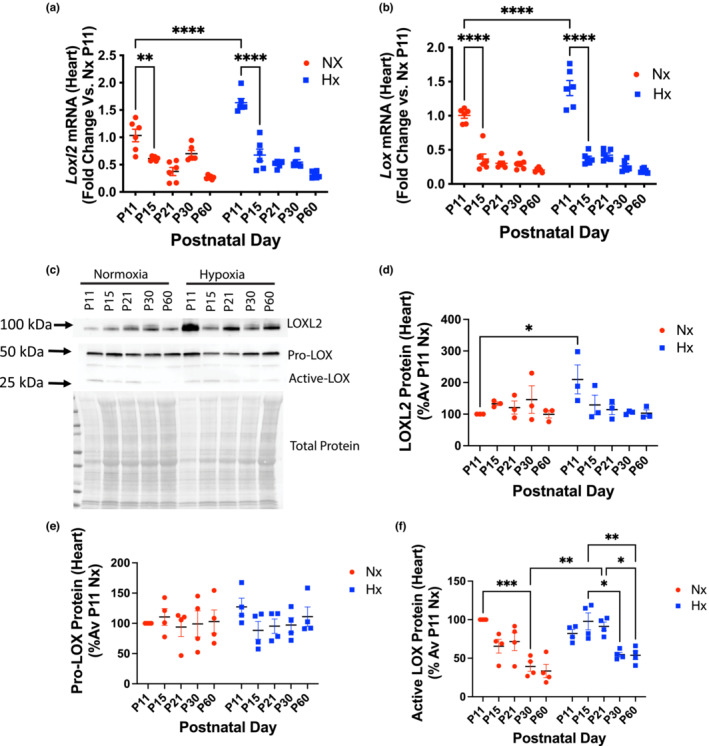
LOXL2 mRNA and protein levels decrease rapidly in the heart after neonatal hypoxia exposure is terminated. (a) *Loxl2* and (b) *Lox* gene expression in the heart with neonatal hypoxia exposure (Hx; fold change vs. normoxia (Nx) P11 hearts with 18S as housekeeping gene; 3 males, 3 females in each group). (c) Representative Western blots of LOXL2, Pro‐LOX, and active‐LOX protein levels in the ECM fraction of the heart. Densitometry analysis of (d) LOXL2, (e) Pro‐LOX, and (f) Active LOX. For Western blots, *n* = 4–6; equal numbers of males and females; *p < 0.05, **p < 0.01, ***p < 0.001, ****p < 0.0001 by 2‐way ANOVA with Bonferroni post hoc analysis. Nx, normoxia, Hx, neonatal hypoxia from P1‐P11.

In the heart, both *Lox* and *Loxl2* gene expression were significantly higher at P11 in the neonatal hypoxia‐exposed group when compared to normoxic counterparts and rapidly decreased with age in both groups (Figure 5a,b). *Loxl2* gene expression was restored to levels similar to normoxic controls by P15 (Figure 5a). Similarly, extracellular matrix (ECM)‐associated LOXL2 protein expression was markedly higher in the P11 hypoxia‐exposed group when compared to normoxic age‐matched controls (Figure 5c,d). ECM LOXL2 protein levels were similar in the two groups from P15‐P60 (Figure 5d). ECM LOXL2 protein abundance did not increase with age in the heart (Figure 5d). Pro‐LOX levels did not change with age or hypoxia (Figure 5e), while active LOX in the ECM decreased rapidly with age in the both groups (Figure 5f) in the ECM.

### 3.3 Arterial stiffening is not limited to the systemic circulation

**FIGURE 6:**
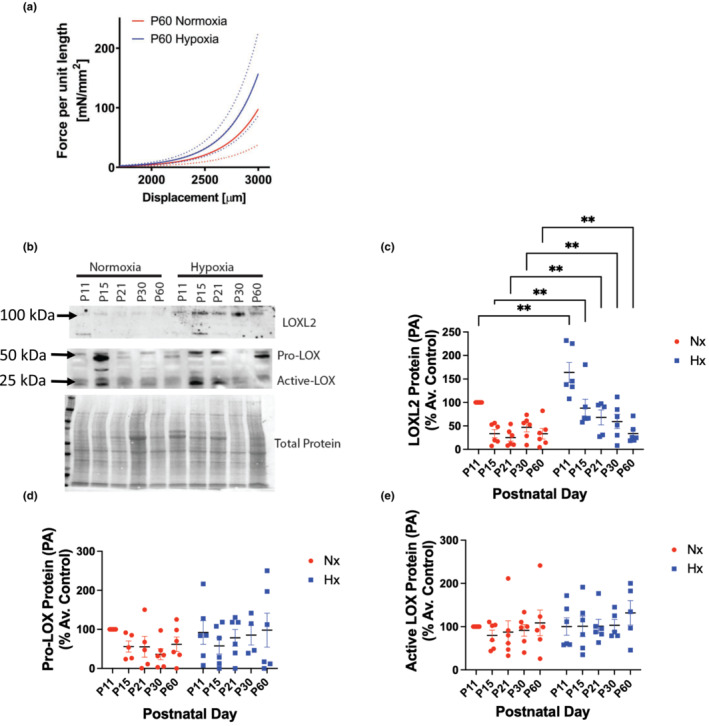
Vascular stiffening and LOXL2 expression of the pulmonary artery after neonatal hypoxia exposure. (a) Tensile testing of pulmonary arteries of 60 day old mice exposed to postnatal hypoxia until day 11 compared with their normoxic littermate controls (*n* = 6, 3 males and 3 females; *p* < 0.0001). (b) Representative Western blots of LOXL2, Pro‐LOX, and active‐LOX protein levels. Densitometry analysis of (c) LOXL2, (d) Pro‐ LOX, and (e) Active LOX. *n* = 4–6 for Western blots; equal numbers of males and females. *p < 0.05, **p < 0.01, ***p < 0.001, ****p < 0.0001 by 2‐way ANOVA with Bonferroni post hoc analysis. Nx, normoxia, Hx, neonatal hypoxia from P1‐P11.

We next evaluated whether arterial stiffening occurs in other large compliance vessels or whether it is limited to the aorta. To this end, we examined the pulmonary artery (PA) and lung, as the pulmonary system is highly sensitive to hypoxia. Mice exposed to neonatal hypoxia had significantly stiffer PAs in early adulthood (P60; Figure 6a). In the PA, LOXL2 protein decreased rapidly with age in the normoxia group; this was delayed in the neonatal hypoxia‐exposed group (Figure 6b,c). LOXL2 expression in the hypoxia group was higher at every age examined when compared to age‐matched normoxia controls. Pro‐ and active LOX were similar with age and hypoxia. There was no change in either Pro‐LOX or active‐LOX protein abundance (Figure 6b,d,e).

**FIGURE 7:**
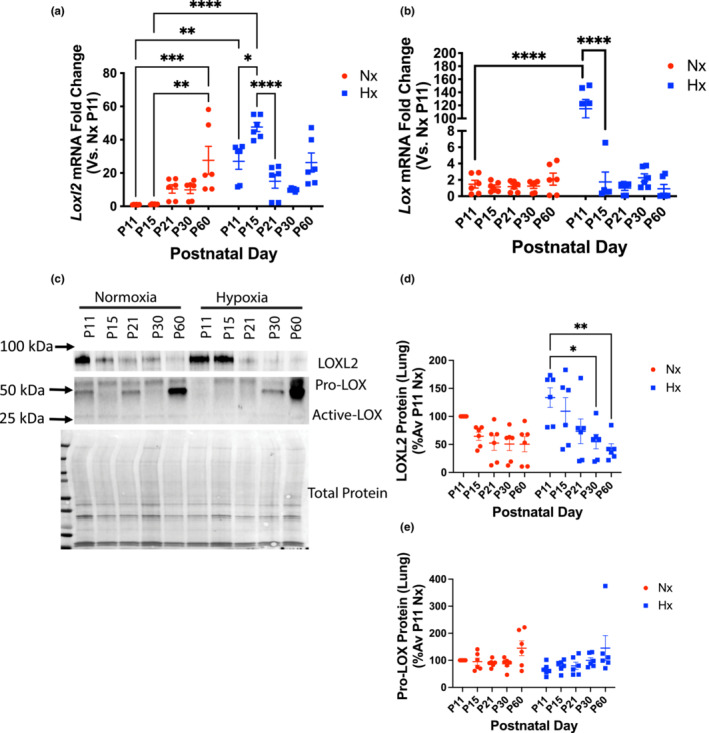
The time course of LOXL2 mRNA and protein levels in the lungs following neonatal hypoxia exposure is distinct from the changes seen in isolated pulmonary arteries. (a) *Loxl2* and (b) *Lox* gene expression in the lung of hypoxic neonates and normoxic littermate controls (fold change vs. P11 normoxia; 18S used as housekeeping gene; *n* = 6 per group; 3 male and 3 female; *p < 0.05, **p < 0.01, ***p < 0.001, ****p < 0.0001 by 2‐way ANOVA with Bonferroni post hoc analysis). (c) Representative Western blots of LOXL2 and Pro‐LOX levels in the lung; active‐LOX band was not detected. Densitometry analysis of (d) LOXL2 and (e) LOX protein in the ECM of lungs. *n* = 4–6 for Western blots, equal numbers of males and females; *p < 0.05, **p < 0.01, ***p < 0.001, ****p < 0.0001 by 2‐way ANOVA with Bonferroni post hoc analysis. Nx, normoxia, Hx, neonatal hypoxia from P1‐P11.

In the lung, *Loxl2* gene expression was elevated at P11 when compared to age‐matched normoxic controls (Figure 7a), remained elevated until P15 (Figure 7a), and returned to levels similar to normoxia by P21 (Figure 7a). Interestingly, an age‐dependent increase in *Loxl2* occurred at P60 in the normoxic mouse lungs (Figure 7a). *Lox* gene expression similarly was increased at P11 in the neonatal hypoxia‐exposed group compared with normoxia controls and rapidly decreased to levels similar to age‐matched normoxic controls. There were no age‐dependent changes in the normoxia group (Figure 7b). As expected, LOXL2 protein abundance in the lung ECM decreased rapidly with age in the normoxia group. The loss of LOXL2 protein with age was delayed in the neonatal hypoxia‐exposed group (Figure 7c,d). No differences were noted in the expression of pro‐LOX with age or hypoxia, and active LOX was not detected (Figure 7e).

### 3.4 Arterial stiffening is accelerated in adulthood in hypoxia‐exposed neonates

**FIGURE 8:**
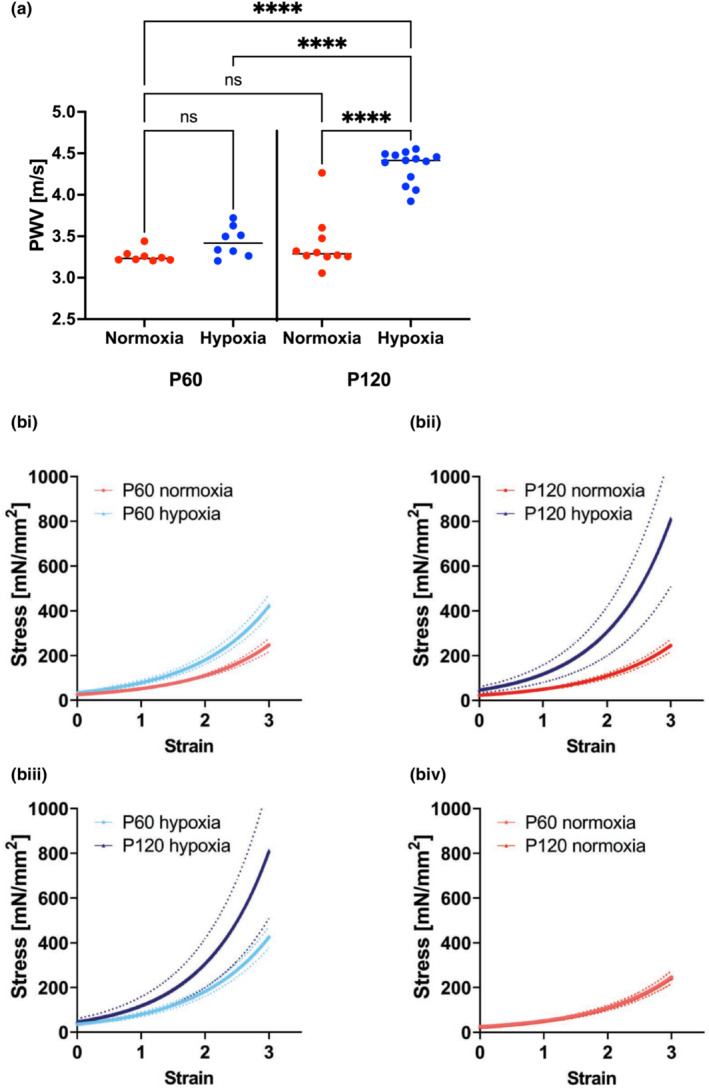
Vascular stiffening during aging occurs at an accelerated pace in adult animals after neonatal hypoxia exposure. (a) Aortic pulse wave velocity (PWV) of hypoxic neonates compared with normoxia controls in early adulthood (P60 and P120) (*n* = 10, 5 male and 5 female; *p* < 0.0001, P120 hypoxia vs. all other groups by 1‐Way ANOVA). (b) Tensile testing of mouse aortas comparing stiffness of normoxic vs. hypoxic mice at P60 (bi) and at P120 (bii); (biii) hypoxic mice at P60 vs. P120 (*n* = 20, 10 male and 10 female; *p* < 0.0001) and (biv) normoxic mice at P60 vs. P120 (*n* = 20, 10 male, 10 female; *p* > 0.05).

Finally, in a subset of animals, we evaluated the progression of aortic stiffness from P60 to P120 of adult life. Both PWV (Figure 8a) and passive stiffness (Figure 8bi–iv) were significantly higher at P120 compared with their age‐matched normoxia controls (Figure 8bii). Moreover, animals exposed to neonatal hypoxia exhibited a further increase in PWV and passive stiffness from P60 to P120 (Figure 8biii); this was absent in normoxic controls (Figure 8biv).

## 4 DISCUSSION

In this study, we examined the effects of neonatal hypoxia and its resolution by term equivalent age on vascular stiffening in adulthood. Aging is typically associated with increased PWV that precedes the manifestation of isolated systolic hypertension. Prior studies focused on aging without co‐morbidities in C57Bl/6 mice showed that arterial stiffening measured as elevated PWV is significant by ~8 months of age (P240) (Steppan et al., 2019). Mice exposed to neonatal hypoxia had higher PWV and passive modulus at P60 with a further increase by P120, without a change in blood pressure, when compared to normoxic controls. The elevated stiffness without BP changes in the neonatal hypoxia group were similar to those observed in middle‐aged mice during aging (Steppan et al., 2019; Wang, Chen, et al., 2021), supporting the hypothesis that exposure to hypoxia during the neonatal period accelerates arterial aging. This is of particular interest, as prior clinical studies have clearly shown that arterial stiffening in childhood and adolescence due to obesity, diabetes, and congenital heart disease is predictive of early onset CVD in adulthood (Dangardt et al., 2013; Hacker et al., 2018; Madsen et al., 2021; Urbina et al., 2010). Thus, in the broader context, our study shows that early exposure to hypoxia is another cause of early arterial stiffening that augments the risk of cardiovascular disease in early adulthood. Moreover, hypoxia in the neonatal period induced arterial remodeling in the pulmonary vasculature as well, suggesting that the effects may be global with regard to stiffening of the large compliance vessels. Interestingly, the changes observed in hypoxic neonates were not confined to the vasculature and extended to the heart as well. Echocardiography revealed a dilated cardiomyopathy occurring as early as P60 in mice exposed to neonatal hypoxia as evidenced by an enlarged left ventricular cavity without signs of increased ventricular mass. Cardiac output, however, was similar in both groups, suggesting that the dilated cardiomyopathy and the decreased ejection fraction were compensated at baseline (e.g., without exertion) by an increased stroke volume. The absence of concentric myocardial hypertrophy despite the increased afterload posed by stiffer vessels suggests that the changes we observed in the heart were either intrinsic to the heart and the compliance vessels or those changes were secondary to a maladaptive response due to the increased afterload.

Both vascular smooth muscle cell dysfunction and vascular ECM remodeling can contribute to the elevated PWV/mechanical stiffening of the aorta. No differences were noted in vasoconstriction, endothelial‐dependent vasorelaxation, or endothelial‐independent vasorelaxation of the aorta in the hypoxic mice at P60. To evaluate whether ECM remodeling could be the underlying mechanism driving stiffening, we determined the expression of LOX and LOXL2 in the ECM of the aorta. The age‐related increase in ECM abundance of LOXL2 was accelerated in the neonatal hypoxia‐exposed group compared with normoxic counterparts, and reached significance at P60. Taken together, these findings suggest that exposure to hypoxia during the neonatal period results in matrix remodeling as a primary cause of accelerated aortic stiffening in early adulthood. In the heart, a remarkable increase in *Lox* and *Loxl2* gene expression was observed at P11 in the hypoxia group, but the magnitude of increase in both LOX and LOXL2 protein abundance in the ECM was modest in comparison. This suggests either a post‐transcriptional regulation of LOXL2 protein expression or a rapid protein degradation/turnover in the neonatal hypoxia‐exposed group. The precise mechanism that governs LOXL2 protein abundance remains to be elucidated. Overall, these data support a role of LOXL2 in accelerated aortic aging in adulthood.

We next determined whether the changes noted in the aorta and heart occur elsewhere in other organ systems. Indeed, PA stiffness was elevated in the neonatal hypoxia‐exposed group at P60. Unlike the aorta, where neonatal hypoxia exposure led to an acceleration in the age‐associated increase in LOXL2 protein, in the PA, LOXL2 expression is sustained at a higher level at all ages examined in the hypoxia group when compared to the normoxia group (i.e., delayed decay of LOXL2 protein abundance with age). In the lung, both *Lox* and *Loxl2* mRNA expressions were elevated. As in the heart, the magnitude of increase in LOXL2 abundance was modest in comparison, and the loss of LOXL2 abundance with age was delayed. *Lox* gene regulation appears to be directly mediated by hypoxia in the lung, as cessation of hypoxia at P11 led to the rapid restoration of *Lox* mRNA to levels similar to normoxic counterparts. The lack of change in LOX protein abundance despite the strikingly elevated *Lox* mRNA level in the P11 hypoxia group suggests post‐transcriptional regulation of LOX protein expression or an accelerated degradation of LOX protein. Taken together, the lung and PA data support a role for elevated levels of LOXL2 in the pulmonary vasculature and lung with neonatal hypoxia exposure.

The trends of LOX and LOXL2 protein expression in the normoxic group are consistent with prior studies that showed that LOX and LOXL2 are important during development and the expression of these proteins declines rapidly after birth. (Bignon et al., 2011; de Jong et al., 2019; Hornstra et al., 2003; Kumarasamy et al., 2009; Steppan et al., 2019) Other studies have also revealed hypoxia‐induced activation of LOX and LOXL2, particularly in the lung. Thus, the upregulation of *Lox* and *Loxl2* mRNA at P11 in the hypoxia group is consistent with the literature. Intriguingly, in the lung, *Loxl2* mRNA remains elevated even at P15, 4 days after cessation of hypoxia. The mechanisms could include either sustained transcription or delayed degradation and remain to be confirmed.

Of clinical relevance, with improved neonatal care there is a growing population of adults who were born prematurely and are reported to have increased blood pressure and increased risk of hypertension (de Jong et al., 2012; Johansson et al., 2005; Skudder‐Hill et al., 2019). While there is greater recognition of this emerging at‐risk population, timely therapeutic interventions, and preventive measures still lag behind in part because the mechanistic perturbations in the cardiopulmonary system due to preterm birth remain poorly delineated. Understanding mechanisms of developmental remodeling and identification of time‐specific intervention, for example, via targeting LOXL2, in this vulnerable population will ensure prevention of early development of cardiovascular disease.

In conclusion, this study shows that exposure to hypoxia in early life accelerates arterial stiffening with aging. LOXL2 plays a more significant role than the prototypical LOX in hypoxia‐induced vascular remodeling and stiffening in both the systemic and pulmonary circulations. As the hypoxia model causes global hypoxia, LOXL2‐mediated vascular remodeling likely occurs in other organ systems and this remains to be tested. The specific mechanism by which LOXL2 mediates arterial stiffening in the aorta versus PA appears to be distinct. In the aorta, neonatal hypoxic exposure accelerated the age‐dependent increase of LOXL2 protein expression, whereas in the PA, it sustained the expression of LOXL2 protein at a higher level for a longer duration in early age when compared to the control normoxia group. Thus, the adult onset of cardiovascular disease noted with neonatal chronic hypoxia is likely related to accelerated arterial stiffening mediated by LOXL2. Future studies using LOXL2 inhibition and/or LOXL2 depletion models will delineate specific mechanisms of LOXL2 activation and its role in driving early onset of arterial stiffening in response to neonatal hypoxia.

## AUTHOR CONTRIBUTIONS

Jochen Steppan, Kavitha Nandakumar, and Huilei Wang performed the experiments, analyzed the data, and drafted the manuscript. Rosie Jang, Logan Smith, Sara Kang, William Savage, Maria Bauer, Rira Choi, Travis Brady, and Bulouere Princess Wodu performed the experiments and analyzed the data. Susanna Scafidi performed the experiments, analyzed the data, drafted the manuscript, provided the funding, and edited the manuscript. Joseph Scafidi and Lakshmi Santhanam conceived the project, performed the experiments, analyzed the data, drafted the manuscript, provided the funding, edited the manuscript, and approved the final version.

## FUNDING INFORMATION

This work was supported by a NHLBI grant R01HL148112 01 (L.S.), a NHLBI grant K08HL145132 (J.St.), NINDS grants R01NS111230 (S.S.), R01NS110808 (S.S.), R01NS099461 (J.Sc.), and R01NS125652 (J.Sc., S.S.). The hypoxia chamber is supported by the Kennedy Krieger Institute and Johns Hopkins University School of Medicine Clinical Translational Core of the Intellectual Developmental Disabilities Research Center award (5P50HD103538) from the National Institute of Child Health and Human Development (NICHD).

## CONFLICT OF INTEREST STATEMENT

The authors declare no conflicts of interest.

## ETHICS STATEMENT

All vertebrate animal experiments were performed in accordance with relevant guidelines and regulations. All animal protocols were approved by the Institutional Animal Care and Use Committee of Johns Hopkins University.
